# Probabilistic model data of spatial-dependent crashes for ranking risk of road segments

**DOI:** 10.1016/j.dib.2019.104966

**Published:** 2019-12-09

**Authors:** Safaa K. Kadhem, Paul Hewson

**Affiliations:** a Al Muthanna University, Faculty of Administration and Economics, Iraq; b University of Plymouth, School of Computing and Mathematics, United Kingdom

**Keywords:** Bayesian inference, Hidden markov models, Model selection, Traffic and transportation

## Abstract

This article presents the databases analyzed and used to evaluate the risk of segment-based roads resulting from traffic crashes for three main motorways in UK from 2010 to 2014. The raw database is collection to many partial data for variables related to compute the crashes rates for each segment. These data were used to develop and select the best Bayesian probabilistic model presented in our research article (Kadhem et al., 2018) [1]. The data provided in this article would be an important source for studies that require evaluating statistical models and also to improve and develop the plans of traffic safety.

**Table:** Specifications of Table

Subject area	Statistics and probability
More specific subject area	Bayesian modeling for traffic crashes
Type of data	Figures, Excel files
How data was acquired	Sorted out from raw crash records
Data format	Raw and analyzed data
Experimental Factors	Road segments crashes data were extracted from raw crash records and sorted out by their riskiness
Experimental features	Road segments were ranked, with respect to their risk, as the highest dangerous based on the highest accidents rates probabilities
Data source location	Department for Transport, UK
Data accessibility	Data with this article

**Table:** 

**Value of the Data** • The provided crash data give illustrative picture about the accidents size that occur in some motorways network in the UK. • Novel applications that involve probability and modeling spatial - dependent crashes to determine the risk each segment road based on the data provided in this article. • Crashes data were spatially collected for segment-based motorway, hence, determining the highest and lowest risk segment to be under studying and focusing by the related official managements. • The data provides indicators to the most safe segments according to the probabilities derived of the low frequencies crashes. • The database consider an important source for studies interested in the analysis and development of traffic safety.

## 1 Data

The databases presented in this article were used to develop and select an optimal probability model, suggested by Kadhem et al., 2018 [1], to determine the states of traffic road riskiness in three motorways in the UK which are: the motorway M5 with 52 sections road, motorway M6 with 90 sections and motorway M42 with 21 sections.

The raw data files (reads in Excel format) were presented in Tables 1–3, respectively, which are deposited at in Supplementary data.

The data reported in this data in brief article (spreadsheets in Supplementary data) were used to develop and select the optimal probability model, suggested by Kadhem et al., 2018 [1], to determine the states of riskiness. The occurred crashes count were recorded as a point process for each segment of road, and those occurring more likely near junctions, over a five-year period from year 2010–2014 in three motorways in the UK which are: the motorway M5 with 52 sections road, M6 with 90 and motorway M42 with 21 sections. Generally, the raw data of each motorway (spreadsheets in Supplementary data) comes from two sources. The data of first sources, obtained from the Department for Transport as an Open Government Archive (OGA) [2], is related to the traffic safety characteristics which are: segment label, crash location, Coordinate Point (CP), Length of segment (L), Annual Average Daily Traffic flow (AADT). While, the data of first sources, which are the crashes count (**y**), obtained from the road traffic counts archive [3].

## 2 Experimental design, materials and methods

**Fig. 1:**
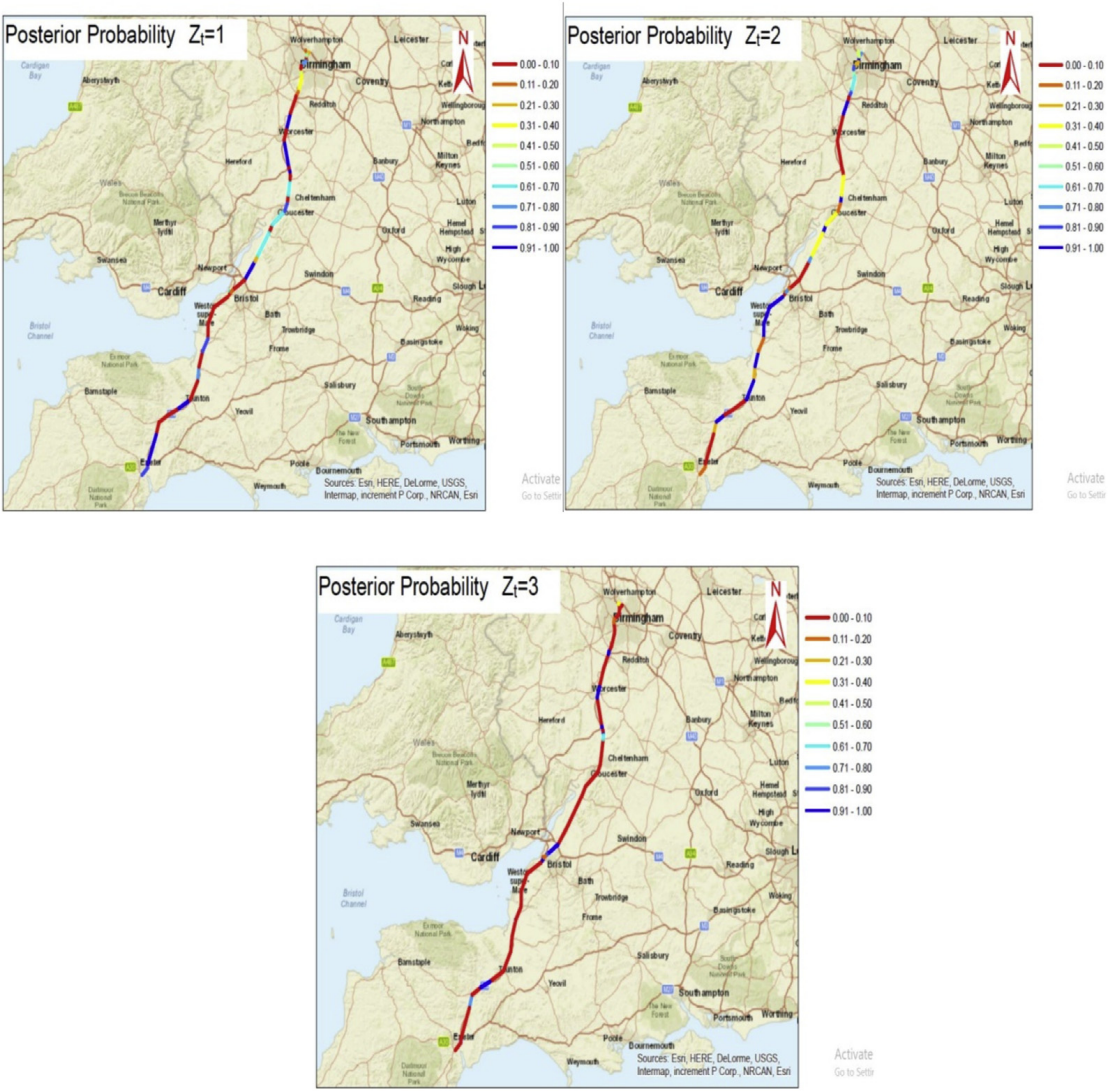
The spatial probabilities mapped at segment level for the M5 motorway.

**Fig. 2:**
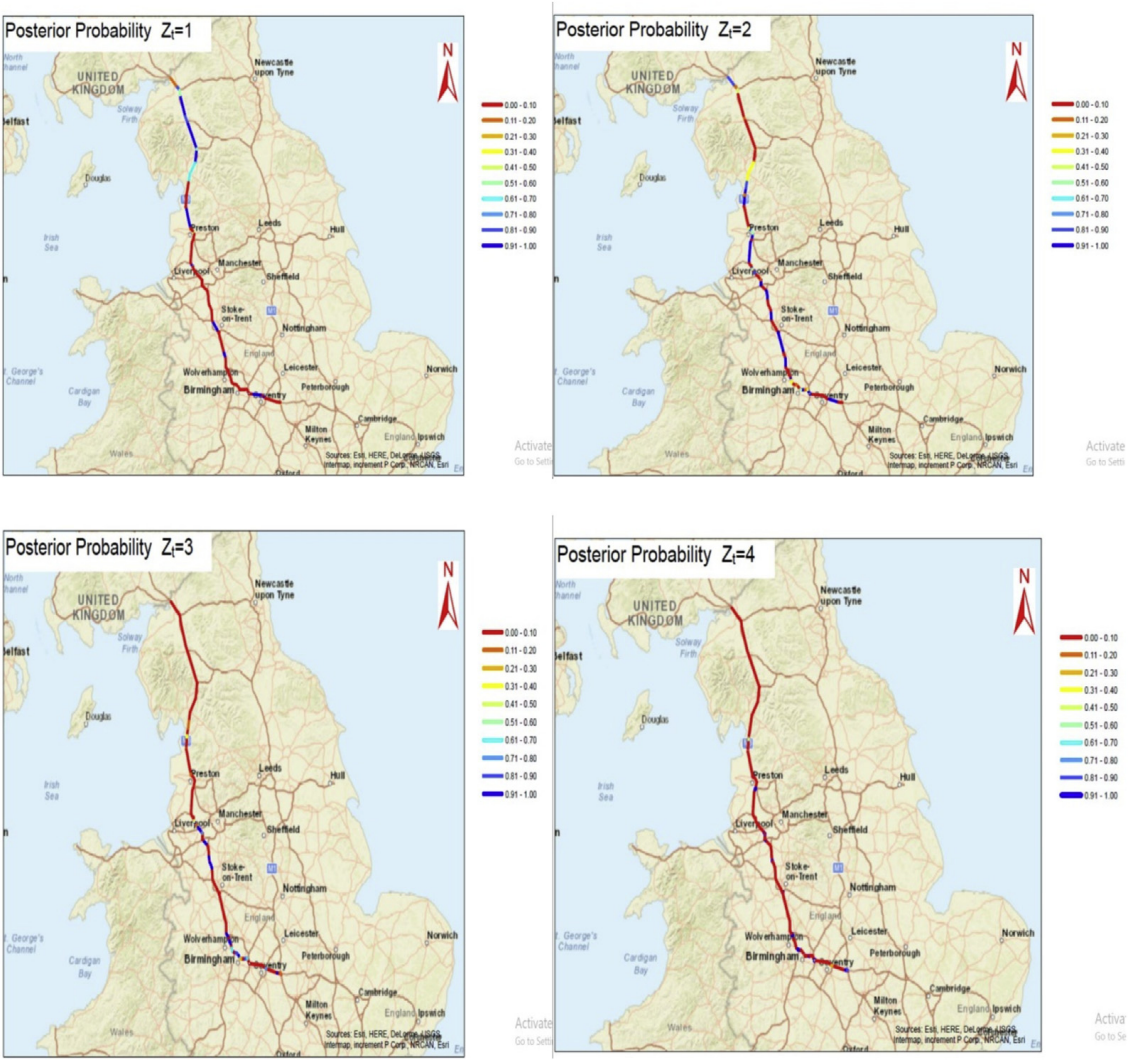
The spatial probabilities mapped at segment level for the M6 motorway.

**Fig. 3:**
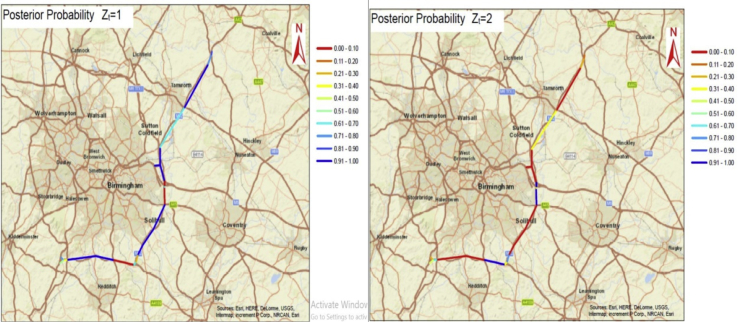
The spatial probabilities mapped at segment level for the M42 motorway.

The processing process of raw data is done through two stages. In the first stage, we compute the expected crash rates (as shown in seventh column of spreadsheets in Supplementary data) which is based on the data of road traffic counts **y** [3] listed in the sixth column of spreadsheets in Supplementary data. In the second stage, we obtain the spatial-based classification probabilities of the hidden states from our model, as shown in the last columns of each motorway in spreadsheets in Supplementary data, based on traffic characteristics given columns from 1 to 6 in spreadsheets in Supplementary data. The Fig. 1, Fig. 2, Fig. 3 show the spatial-based classification probabilities of the hidden states for each motorway which were plotted and mapped using the Arc Geographic Information System (ArcGIS) [4].
